# A modified microchip-based flow chamber system for evaluating thrombogenicity in patients with thrombocytopenia

**DOI:** 10.1186/s12959-020-00244-9

**Published:** 2020-10-30

**Authors:** Bengo Atari, Takashi Ito, Tomoka Nagasato, Tomoko Ohnishi, Kazuya Hosokawa, Tomotsugu Yasuda, Ikuro Maruyama, Yasuyuki Kakihana

**Affiliations:** 1 Department of Emergency and Intensive Care Medicine, Kagoshima University Graduate School of Medical and Dental Sciences, Kagoshima, Japan; 2 Department of Systems Biology in Thromboregulation, Kagoshima University Graduate School of Medical and Dental Sciences, 8-35-1 Sakuragaoka, Kagoshima, 890-8544 Japan; 3 Research Institute, Fujimori Kogyo Co., Ltd., Yokohama, Japan

**Keywords:** Thrombocytopenia, Platelet transfusion, Bleeding, Flow chamber, Total Thrombus-formation analysis system (T-TAS)

## Abstract

**Background:**

In the intensive care unit (ICU), patients with thrombocytopenia are at high risk for bleeding and should be assessed for their thrombogenic potential. However, the analytical conditions of conventional hemostatic tests are unsuitable for the evaluation of low-platelet samples. Here we aimed to establish suitable analytical conditions with the Total Thrombus-formation Analysis System (T-TAS) for quantitative assessment of thrombogenic potential in patients with thrombocytopenia and to investigate how T-TAS values relate to bleeding symptoms and the effects of platelet transfusion.

**Methods:**

Modified chips with a different chamber depth were developed for the analysis of low-platelet samples in the T-TAS. We included 10 adult patients admitted to the ICU of Kagoshima University Hospital who required platelet transfusion. Patients were divided into major and minor bleeding groups according to their bleeding scale before platelet transfusion. The thrombogenic potential of these patients before and after platelet transfusion was assessed with hemostatic function tests, including rotational thromboelastometry, multiplate aggregometry, and the T-TAS.

**Results:**

Analysis of low-platelet samples revealed that, compared with the conventional chip (80-μm-deep chamber), the modified chip (50-μm-deep chamber) achieved higher sensitivity in detecting elevation of flow pressure caused by growth of an occlusive thrombus in the T-TAS analytical chamber. All patients in the minor bleeding group retained thrombogenic potential that occluded the modified chip (occlusion time 16.3 ± 3.3 min), whereas most patients in the major bleeding group were unable to occlude the modified chip during the 30-min measurement (*P* <  0.01). The recovery of thrombogenic potential after platelet transfusion was confirmed with the T-TAS and correlated with the function, rather than the count, of transfused platelets. Among all evaluated parameters in hemostatic function tests, only the T-TAS showed significant differences in occlusion time and area under the curve both between the minor and major bleeding groups and between pre- and post-platelet transfusion.

**Conclusions:**

We developed a modified microchip-based flow chamber system that reflects the hemostatic function of patients with thrombocytopenia.

## Background

Patients in the intensive care unit (ICU) often experience thrombocytopenia. Causes of thrombocytopenia include loss of platelets resulting from hemorrhage, dilution resulting from fluid resuscitation, consumption resulting from platelet adhesion to the vascular wall or extracorporeal devices, and insufficient production resulting from hematopoietic disease or adverse drug effects [1–3]. To manage thrombocytopenia, it is important to identify and eliminate its causes and, if necessary, transfuse platelets to stabilize hemostasis and hemodynamics.

Practice criteria for platelet transfusion are controversial. Platelets may be transfused to patients with thrombocytopenia during treatment of hematopoietic disease, before insertion of a central venous catheter, before diagnostic lumbar puncture, and before surgery. Practice criteria are not standardized and depend to a large extent on the circumstances mentioned above [4–8]. Randomized controlled trials comparing patients with hematopoietic disease who received prophylactic platelet transfusion when platelets fell to < 10,000/μL with those who received platelet transfusion after bleeding symptoms appeared found that the former group underwent transfusions more frequently. However, these patients experienced fewer bleeding events, suggesting the benefit of prophylactic platelet transfusion [9–11]. The frequency of bleeding events in patients with hematopoietic malignancies does not significantly differ when the trigger value for platelet transfusion is 10,000/μL versus 20,000/μL [12–14]; therefore, a platelet count of 10,000/μL has been proposed as the transfusion trigger value. However, platelet counts do not significantly correlate with bleeding events [15], suggesting that indicators more closely related to bleeding symptoms may be more appropriate for assessing the need for platelet transfusion.

Thromboelastometry and multiplate impedance aggregometry are widely used for analyzing hemostatic function. Thromboelastometry, which monitors changes in the viscoelasticity of whole blood during formation of the fibrin clot, is mainly used to evaluate hemostatic function during cardiovascular surgery [16–18]. Multiplate impedance aggregometry, which monitors the increase in electrical resistance between electrodes caused by platelet aggregation, is used to evaluate the efficacy of antiplatelet drugs [19–21]. These methods are employed worldwide as point-of-care hemostatic function tests using whole blood. However, the ability of these tests to evaluate hemostatic function in patients with thrombocytopenia and to determine the requirements for platelet transfusion have not been established.

We developed the Total Thrombus-formation Analysis System (T-TAS), which comprehensively evaluates hemostatic function under conditions similar to those of thrombus formation in vivo [22]. The T-TAS monitors the elevation in flow pressure, which reflects the growth of a platelet- and fibrin-rich thrombus in the flow chamber, which is embedded in an analytical chip. Recent studies have suggested that T-TAS values are a significant predictor of bleeding events [23, 24]. However, as with other conventional hemostatic function tests, the analytical conditions of the T-TAS are not suitable for evaluating hemostatic function in patients with thrombocytopenia. Here we developed analytical conditions suitable for the evaluation of hemostatic function of low-platelet samples by adjusting the depth of the T-TAS flow chamber. We further determined how the values acquired with the modified T-TAS were related to bleeding symptoms as well as the effects of platelet transfusion in patients with thrombocytopenia.

## Methods

### Blood sampling

This single-center observational study was approved by the Ethics Committee of Kagoshima University Hospital (approval no. 170178–2). The study was conducted in compliance with the Declaration of Helsinki, and written informed consent to participate was obtained from patients or their close relatives. We included 10 adult patients admitted to the ICU of Kagoshima University Hospital between November 2017 and October 2019 who required a platelet transfusion. We excluded patients administered drugs that affect the hemostatic system, such as antiplatelet agents or anticoagulants. The use of anticoagulants for the maintenance of arterial lines or extracorporeal devices was permitted. The need for platelet transfusion was determined in accordance with our standard clinical practice, independent of the present study.

Blood was drawn from the radial arterial line before and after platelet transfusion. The samples were anticoagulated with EDTA (Becton Dickinson Co., Fukushima, Japan), 3.2% sodium citrate (Terumo, Tokyo, Japan), or hirudin (Roche Diagnostics GmbH, Mannheim, Germany) and were used for blood cell counts, thromboelastometry, T-TAS, and multiplate aggregometry. After platelet transfusion, platelet concentrates remaining in the transfusion tube were collected and analyzed for their thrombogenic potential.

### Assessment of bleeding scale

Before platelet transfusion, bleeding symptoms were assessed with the modified WHO bleeding scale [4]. Grades 0, 1, 2, 3, and 4 corresponded to minimum, minor, moderate, severe, and debilitating bleeding, respectively. Patients were divided into two groups according to their bleeding scale before platelet transfusion: the minor bleeding group, which included patients with a bleeding grade ≤ 1, and the major bleeding group, which included patients with a grade ≥ 2.

### Laboratory tests

Platelet counts were measured with an XN-9000 automated blood cell analyzer (Sysmex, Kobe, Japan). General coagulation tests, including measurement of prothrombin time (PT), activated partial thromboplastin time (APTT), and fibrinogen, were performed with the Automated Coagulation System-CP3000 (Sekisui Medical, Tokyo, Japan) or a STACIA (LSI Medience, Tokyo, Japan).

### Rotational thromboelastometry (ROTEM)

Blood anticoagulated with sodium citrate was used for ROTEM (Instrumentation Laboratory). Tissue factor-induced blood coagulation (EXTEM) and ellagic acid-induced blood coagulation (INTEM) were analyzed with ROTEM according to the protocol recommended by the manufacturer, and clotting time and maximum clot firmness were evaluated.

### Multiple electrode aggregometry

Blood anticoagulated with hirudin was used for multiple electrode aggregometry with a Multiplate Analyzer (Roche). Platelet aggregation induced by collagen, adenosine diphosphate (ADP), thrombin receptor activating peptide-6 (TRAP-6), or ristocetin was analyzed according to the protocol recommended by the manufacturer, and the area under the curve (AUC) was evaluated.

### T-TAS analysis

Blood anticoagulated with sodium citrate was used for T-TAS analysis (Fujimori Kogyo, Tokyo, Japan). Calcium chloride and corn trypsin inhibitor were added at the start of the measurement. In some experiments, platelet concentrates (240 μL) mixed with pooled normal plasma (240 μL) (George King Bio-Medical, Inc., Overland Park, KS, USA) and a reagent mix (20 μL) containing calcium chloride, corn trypsin inhibitor, aprotinin, and heparan sulfate were used for T-TAS analysis to evaluate the hemostatic function of platelet concentrates. Thrombus formation in the flow chamber, which was coated with tissue factor and collagen, was analyzed according to the protocol recommended by the manufacturer, and occlusion time and the AUC were evaluated. For the analysis of low-platelet samples, newly developed flow chambers (width, 300 μm; depth, 60 μm or 50 μm) were used instead of conventional flow chambers (width, 300 μm; depth, 80 μm). The flow rate was set at 10 μL/min, which corresponds to initial wall shear rates of approximately 1500, 1100, and 600 s^− 1^ in the 50-, 60-, and 80-μm-deep chambers, respectively. The intra-assay coefficient of variation of the AUC was 1.24% when using conventional flow chambers and whole blood from healthy volunteers [25] .

### Immunofluorescence analysis of flow chambers

Immediately after the T-TAS analysis, immunofluorescence analysis was performed to determine the composition of thrombi formed in the flow chamber [22]. Platelets in unfixed thrombi were labeled with FITC-conjugated mouse anti-human CD41 IgG (Beckman Coulter, Miami, FL, USA) for 15 min in the dark. After fixation with OptiLyse C (Beckman Coulter), fibrin (ogen) was detected by using rabbit anti-human fibrinogen IgG (Dako, Tokyo, Japan) labeled with Alexa Fluor 594 (Invitrogen, Carlsbad, CA, USA) for 30 min in the dark. The nuclei of leukocytes were stained with 4′,6-diamidino-2-phenylindole dihydrochloride (Dojindo, Kumamoto, Japan). The entire image of thrombi formed in the flow chamber was analyzed with a BZ-X700 All-in-One Fluorescence Microscope (Keyence Corp., Osaka, Japan). Although fibrin generation could continue until fixation with OptiLyse C, this had little impact on the results.

### Statistical analysis

The significance of differences between the major and minor bleeding groups was evaluated with the Student *t* test. The significance of differences before versus after platelet transfusion was evaluated with the paired *t* test. Relationships between hemostatic function test values were evaluated with Pearson’s correlation coefficients and *P* values. All statistical analyses were performed with IBM SPSS version 23 (Armonk, NY, USA), and a *P* value < 0.05 was considered to indicate a significant difference.

## Results

### Novel flow chambers for evaluating thrombogenicity of low-platelet samples

**Fig. 1:**
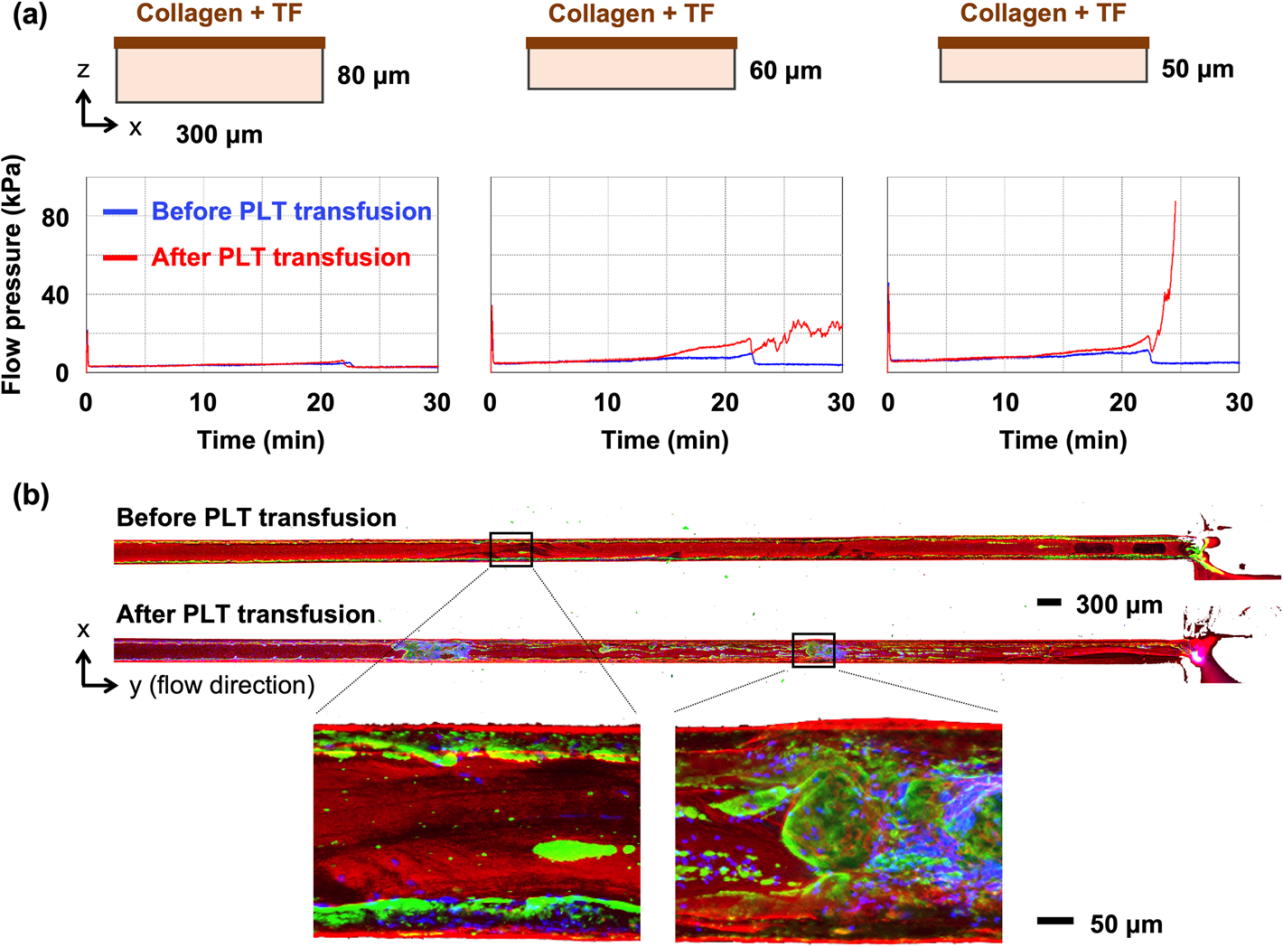
Modified flow chambers for evaluating thrombogenicity of low-platelet samples. **a** Cross-sectional views of the T-TAS flow chamber are shown in the upper panel. The width (x) is 300 μm in all cases and the depth (z) is 80 μm, 60 μm, or 50 μm. The ceiling is coated with tissue factor (TF) and collagen, which are inducers of thrombus formation. Whole blood samples were perfused (10 μL/min) through the flow chamber until flow pressure reached 80 kilopascal (kPa) or for 30 min. Representative waveforms of flow pressure in a patient with thrombocytopenia are shown in the lower panel. Blue and red lines indicate waveforms before and after platelet (PLT) transfusion, respectively. **b** Representative images of thrombi formed in the assay (**a**) are shown. Platelets (green), fibrin/fibrinogen (red), and nuclei of leukocytes (blue) in the flow chambers (50-μm deep) were visualized with the All-in-One Fluorescence Microscope. The y-axis indicates the direction of blood flow. The scale bars in the upper and lower panels indicate 300 μm and 50 μm, respectively

When we used conventional T-TAS chips with a chamber depth of 80 μm to analyze low-platelet samples, the flow pressure did not increase during the 30-min measurement (Fig. 1a). This finding indicates that conventional T-TAS chips are unsuitable for quantitative analysis of hemostatic function in patients who require a platelet transfusion. To detect an elevation in flow pressure in low-platelet samples, we developed modified chips with a chamber depth of 60 μm or 50 μm. When we used the 50-μm chip, the flow pressure increased after platelet transfusion (Fig. 1a). Immunofluorescence analysis revealed that platelet transfusion resulted in enlargement of platelet-rich thrombi covered with a fibrin (ogen) mesh that filled the chamber (Fig. 1b). Large numbers of leukocytes were trapped in the platelet-rich thrombi after platelet transfusion. We subsequently used the 50-μm chips, designated HD chips, instead of the conventional AR chips for the analysis of low-platelet samples in the T-TAS.

### Relationship between bleeding symptoms and values of hemostatic function tests

**Fig. 2:**
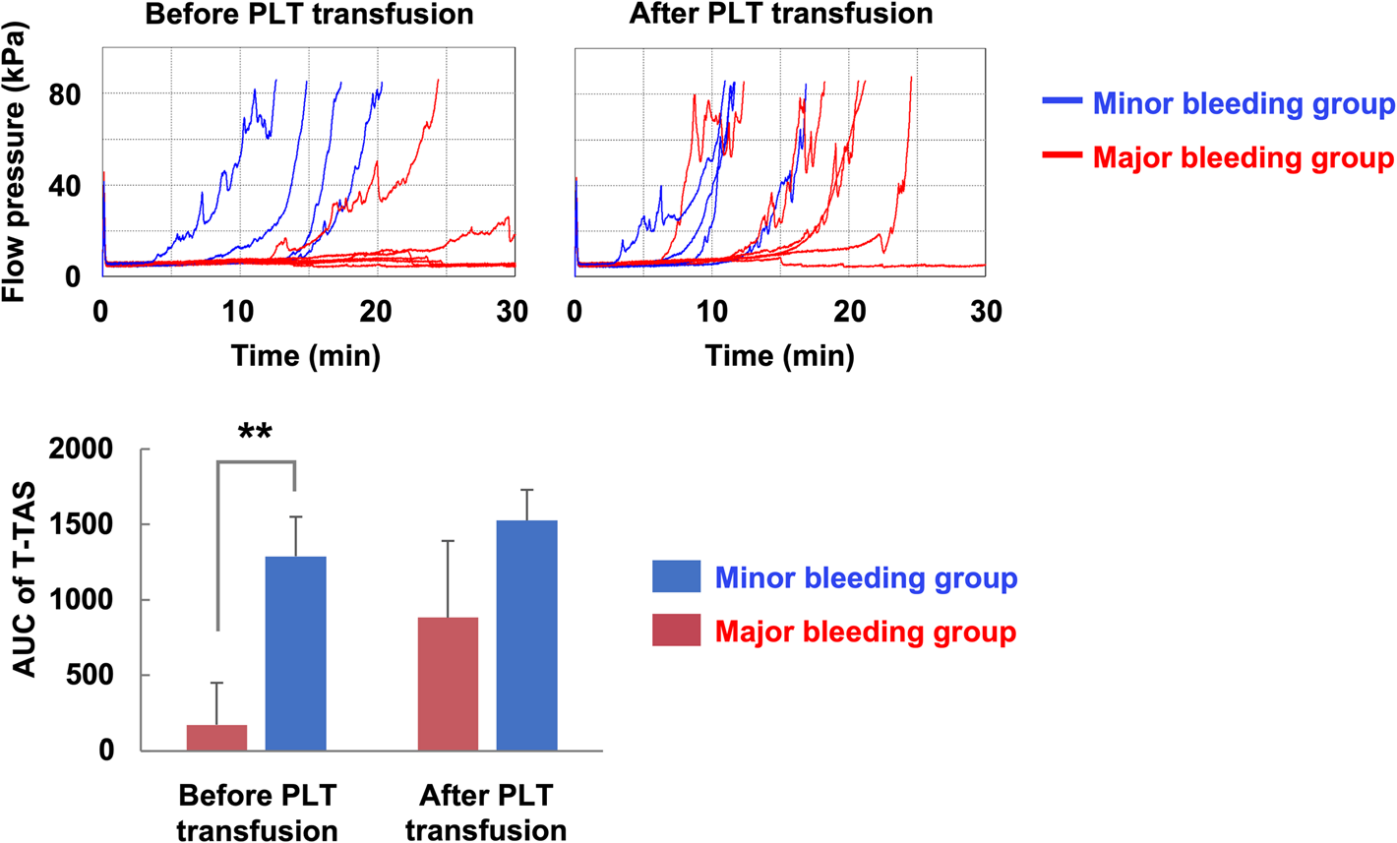
T-TAS discriminates hemostatic function of major bleeding patients from that of minor bleeding patients. Overlays of T-TAS waveforms of 10 patients requiring platelet (PLT) transfusion, upper panel. Blue and red lines indicate waveforms in the minor bleeding (*n* = 4) and major bleeding (*n* = 6) patients, respectively. The area under the curve (AUC) of the T-TAS, expressed as mean ± standard deviation, lower panel. The significance of the difference between the major and minor bleeding groups was analyzed with the Student *t* test. **P < 0.01

We next analyzed the relationship between bleeding symptoms and the values of hemostatic function tests, including ROTEM, the Multiplate Analyzer, and the T-TAS, in 10 patients requiring platelet transfusion (Table 1). Of these, six patients were categorized into the major bleeding group and four were categorized into the minor bleeding group. Most conventional test values other than the EXTEM clotting time did not significantly differ between the major and minor bleeding groups (Table 2). In contrast, the occlusion time and the AUC of the T-TAS HD chips did significantly differ between groups (*P* <  0.01). When the T-TAS HD chip was used, all four patients in the minor bleeding group retained thrombogenic potential to occlude the flow chamber. In contrast, patients in the major bleeding group showed delayed or defective thrombus formation in the T-TAS HD chips (Fig. 2). These findings suggest that T-TAS values were closely related to bleeding tendency and may therefore be useful for quantitative analysis of hemostatic function of patients with thrombocytopenia.

**Table 1:** Patients’ baseline characteristics

Patient	Age	Sex	Underlying disease	Platelet count (10^3^/μL)	Bleeding scale	Sites and duration	Reason for transfusion
1	84	F	Microscopic polyangitis	25	1	Soft tissue, temporal	Prophylactic
2	36	M	SLE	58	3	Oral cavity, persistent	Bleeding tendency
3	52	F	TAFRO syndrome	43	0	None	Before the invasive procedure
4	70	F	Acute liver failure	27	2	Soft tissue, temporal	Bleeding tendency
5	65	M	Alcoholic hepatitis	11	4	GI tract, persistent	Bleeding tendency
6	44	F	Acute pancreatitis	53	1	Soft tissue, temporal	Prophylactic
7	76	M	Sepsis	16	2	Urinary, temporal	Bleeding tendency
8	84	M	SFTS	38	2	Soft tissue, persistent	Bleeding tendency
9	28	M	AML	13	3	Trachea, persistent	Bleeding tendency
10	73	M	Liver abscess	38	0	None	Before the invasive procedure

*F* female; *M* male; *SLE* systemic lupus erythematosus; *TAFRO* thrombocytopenia, anasarca, myelofibrosis, renal dysfunction, and organomegaly; *SFTS* severe fever with thrombocytopenia syndrome; *AML* acute myeloid leukemia; *GI* gastrointestinal

**Table 2:** Patients’ hemostatic values before platelet transfusion

Hemostatic parameter		Major bleeding group (mean ± SD)	Minor bleeding group (mean ± SD)	*P*
General tests	Platelet (10^3^/μL)	27.2 ± 18.2	39.8 ± 11.6	0.259
	PT-INR	1.4 ± 0.5	1.5 ± 0.5	0.733
	PT (sec)	16.3 ± 5.3	17.8 ± 5.7	0.676
	APTT (sec)	71.2 ± 28.7	74.8 ± 41.0	0.872
	Fibrinogen (mg/dl)	414.5 ± 343.0	385.8 ± 439.9	0.910
ROTEM	EXTEM clotting time (sec)	94.5 ± 18.7	64.5 ± 13.4	0.025
	EXTEM MCF (mm)	41.0 ± 5.7	44.3 ± 12.4	0.583
	INTEM clotting time (sec)	299.2 ± 85.9	284.3 ± 55.3	0.768
	INTEM MCF (mm)	39.5 ± 4.4	40.8 ± 12.6	0.824
Multiplate	Collagen-induced aggregation (U)	67.5 ± 51.6	100.5 ± 23.0	0.270
	ADP-induced aggregation (U)	19.3 ± 17.2	42.0 ± 17.7	0.078
	TRAP-induced aggregation (U)	44.3 ± 46.2	69.8 ± 23.2	0.344
	Ristocetin-induced aggregation (U)	8.7 ± 8.4	5.0 ± 3.4	0.439
T-TAS	Occlusion time (min)	> 30	16.3 ± 3.3	< 0.01
	AUC	171.5 ± 305.3	1291.2 ± 298.7	< 0.01

*SD* standard deviation; *PT* prothrombin time; *INR* international normalized ratio; *APTT* activated partial thromboplastin time; *MCF* maximum clot firmness; *ADP* adenosine diphosphate; *TRAP* thrombin receptor activating peptide; *AUC* area under the curve

### Differences in values of hemostatic function tests before versus after platelet transfusion

**Fig. 3:**
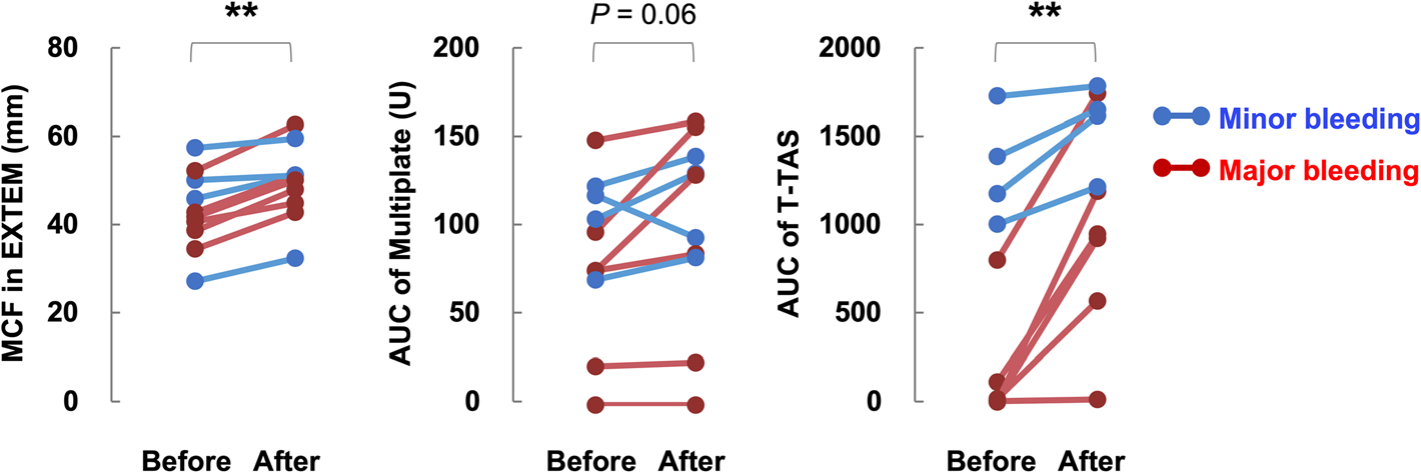
T-TAS detects recovery of hemostatic function after platelet transfusion. Changes in the maximum clot firmness (MCF) in EXTEM, the AUC determined with the Multiplate Analyzer, and the AUCs of T-TAS analysis before and after platelet transfusion. Blue and red lines indicate changes in the minor bleeding (n = 4) and major bleeding (n = 6) patients, respectively. The significance of differences between values before and after platelet transfusion was analyzed with a paired *t* test. **P < 0.01

We next analyzed differences in the values of hemostatic function tests before versus after platelet transfusion. When we measured thrombogenicity with the Multiplate Analyzer, there was no significant difference before versus after platelet transfusion. In contrast, the maximum clot firmness determined with the ROTEM and the thrombogenic potential determined with the T-TAS were significantly higher after platelet transfusion than before transfusion (Fig. 3). Patients with low T-TAS values even after platelet transfusion required repeated platelet transfusions (Table 3).

**Table 3:** Bleeding scale, AUC of T-TAS, and need for platelet transfusion between day 1 and day 7

Patient	Bleeding Scale	AUC of T-TAS		Day 1	Day 2	Day 3	Day 4	Day 5	Day 6	Day 7
		Before	After							
1	1	984.5	1188.1	●						
2	3	128.3	929.9	●						
3	0	1150.1	1572.4	●						●
4	2	27.5	907.4	●				●		
5	4	20.4	1164.9	●		●				
6	1	1351.4	1607.3	●						
7	2	38.3	568.2	●	●	●				
8	2	789.2	1694.2	●					●	
9	3	25.3	34.3	●	●	●	●	●	●	●
10	0	1678.6	1734	●						

● indicates the day requiring platelet transfusion. The requirement for platelet transfusion was determined in accordance with our standard clinical practice, independent of the present study

**Fig. 4:**
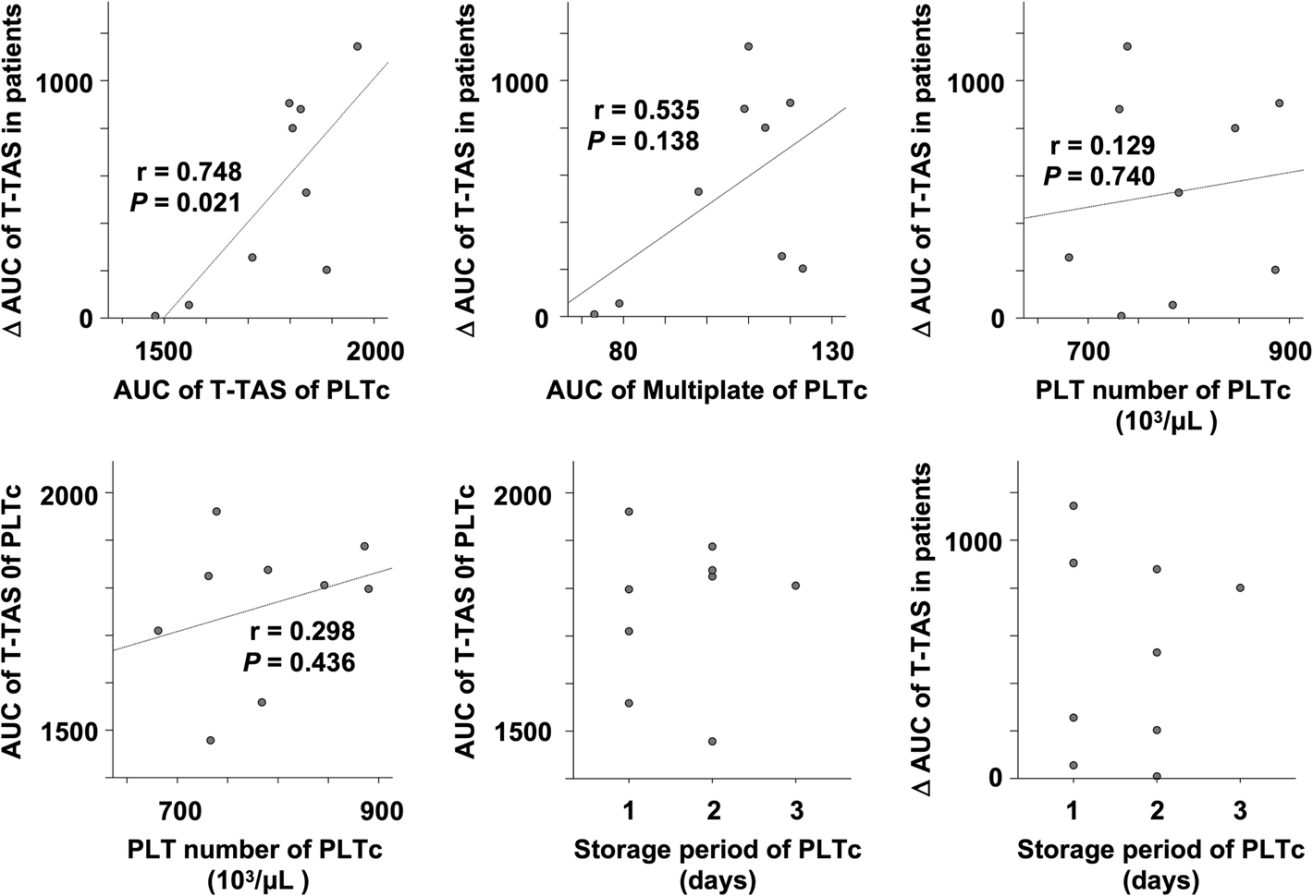
Hemostatic function recovery rate depends on platelet function, not count, in transfused platelet concentrate. (Upper left) Correlation between the difference in the AUC of the T-TAS of blood collected before versus after platelet transfusion (ΔAUC of T-TAS in patients) and the AUC of the T-TAS of platelet concentrate (PLTc); Pearson’s correlation coefficient (r) = 0.748, *P* = 0.021. (Upper middle) Correlation between the ΔAUC of the T-TAS in patients and the AUC of the Multiplate Analyzer; r = 0.535, *P* = 0.138. (Upper right) Correlation between the ΔAUC of the T-TAS in patients and the platelet number of the PLTc; r = 0.129, *P* = 0.740. (Lower left) Correlation between the AUC of the T-TAS of PLTc and the platelet number of the PLTc; r = 0.298, *P* = 0.436. (Lower middle) Relationship between the AUC of the T-TAS and the storage period of PLTc (days between blood donation and transfusion). (Lower right) Relationship between the ΔAUC of the T-TAS in patients and the storage period of PLTc. The data for one of the 10 PLTc were unavailable, and thus nine cases were analyzed

Because the recovery rate of thrombogenic potential after platelet transfusion (ΔAUC of T-TAS) varied from one case to another, we examined factors closely related to this rate. The ΔAUC of the T-TAS in patients significantly correlated with the AUC of the T-TAS of the platelet concentrate, but did not correlate with the number of platelets in the platelet concentrate (Fig. 4, upper panel). The AUC of the T-TAS of platelet concentrates did not correlate with the number of platelets or the storage period of platelet concentrates (Fig. 4, lower panel). These findings indicate that the recovery rate of thrombogenic potential after platelet transfusion may depend on the function, rather than the count, of platelets in the platelet concentrate.

## Discussion

The major findings of this study are as follows. (1) We developed modified T-TAS HD chips suitable for quantitatively evaluating hemostatic function in patients with thrombocytopenia. (2) T-TAS HD chips discriminated between hemostatic function of the major and minor bleeding groups. (3) T-TAS HD chips detected the recovery of hemostatic function following platelet transfusion. (4) The recovery of hemostatic function following platelet transfusion may depend on the function of transfused platelets, rather than the count. The advantages of T-TAS HD chips do not necessarily suggest that the T-TAS is superior to conventional hemostatic function tests, such as those obtained with the Multiplate Analyzer and ROTEM, because suitable analytical conditions for low-platelet samples are indispensable, not only for the T-TAS but also for the Multiplate Analyzer and ROTEM.

It is important to determine whether evaluation of hemostatic function with the T-TAS supports clinical decision-making. For example, the ability of the T-TAS to indicate the need for platelet transfusion should be clarified in the future. Answering this critically important clinical question requires prospective investigation of how the frequency of bleeding events and platelet transfusion differ when criteria based on the T-TAS versus platelet counts are applied. Such interventional studies may not be approved at this time because insufficient data are available to ensure that the T-TAS may be safely used to make clinical decisions. We therefore consider that the present study is a first step in this direction.

Efficient hemostasis requires sufficient numbers of functional platelets as well as coagulation factors [26, 27]. If platelet function is compromised, hemostasis is impaired regardless of platelet count. For example, the platelet counts in patients #8 and #10 were similar, although the thrombogenicity of the former was low according to T-TAS data, and bleeding symptoms were apparent. The risk of bleeding might be more accurately evaluated with a comprehensive analysis of hemostatic function using the T-TAS. This might be an advantage of the T-TAS in comparison with specific tests targeting platelets or coagulation function.

The recovery of thrombogenic potential after platelet transfusion was confirmed in most patients, with the exception of patient #9. Table 3 indicates that patients with a low AUC of the T-TAS after platelet transfusion required additional platelet transfusion within the next few days. Thus, the platelet transfusion in patient #9 on day 1 might have had limited effectiveness. This finding might have resulted partly from the relatively low function of the transfused platelets and the fact that the background disease did not allow recovery of platelet count in this patient.

The 10 patients tested in the present study had different underlying diseases. It is important to consider whether the cut-off values must be changed according to the underlying disease when making clinical decisions. Although it is possible that thrombogenic potential can be uniformly determined with the T-TAS regardless of underlying disease, the T-TAS is unable to evaluate the vulnerability of blood vessels, which is one factor that contributes to bleeding [28–31]. Thus, the T-TAS may underestimate the risk of bleeding in diseases associated with vascular abnormalities. Detailed analysis of each underlying disease will be required in the future.

## Conclusions

We developed a modified microchip (HD chip)-based flow chamber system suitable for evaluating the thrombogenicity of patients with thrombocytopenia. For analysis of blood samples with normal platelet counts, we still recommend using conventional AR chips.

## Data Availability

The datasets used and/or analyzed during the current study are available from the corresponding author on reasonable request.

## Abbreviations

ADP: Adenosine diphosphate
APTT: Activated partial thromboplastin time
AUC: Area under the curve
ICU: Intensive care unit
PT: Prothrombin time
ROTEM: Rotational thromboelastometry
TRAP-6: Thrombin receptor activating peptide-6
T-TAS: Total thrombus-formation analysis system

## References

[1] Smock KJ, Perkins SL (2014). Thrombocytopenia: an update. Int J Lab Hematol.

[2] Rankin JS, Stratton CW (2014). Efficacy of immunomodulation in the treatment of profound thrombocytopenia after adult cardiac surgery. J Thorac Cardiovasc Surg.

[3] Sinkovič A, Majal M (2015). The impact of thrombocytopenia on outcome in patients with acute coronary syndromes: a single center retrospective study. Biomed Res Int.

[4] Kaufman RM, Djulbegovic B, Gernsheimer T, Kleinman S, Tinmouth AT, Capocelli KE, Cipolle MD, Cohn CS, Fung MK, Grossman BJ (2015). Platelet transfusion: a clinical practice guideline from the AABB. Ann Intern Med.

[5] Estcourt LJ, Birchall J, Lowe D, Grant-Casey J, Rowley M, Murphy MF (2012). Platelet transfusions in haematology patients: are we using them appropriately?. Vox Sang.

[6] Liumbruno G, Bennardello F, Lattanzio A, Piccoli P, Rossetti G (2009). Recommendations for the transfusion of plasma and platelets. Blood Transfus.

[7] Slichter SJ. Evidence-based platelet transfusion guidelines. Hematol Am Soc Hematol Educ Program. 2007;2007(1):172–8. 10.1182/asheducation-2007.1.172. 10.1182/asheducation-2007.1.172 18024626

[8] Schiffer CA, Anderson KC, Bennett CL, Bernstein S, Elting LS, Goldsmith M, Goldstein M, Hume H, McCullough JJ, McIntyre RE (2001). Platelet transfusion for patients with cancer: clinical practice guidelines of the American Society of Clinical Oncology. J Clin Oncol.

[9] Murphy S, Litwin S, Herring LM, Koch P, Remischovsky J, Donaldson MH, Evans AE, Gardner FH (1982). Indications for platelet transfusion in children with acute leukemia. Am J Hematol.

[10] Wandt H, Schaefer-Eckart K, Wendelin K, Pilz B, Wilhelm M, Thalheimer M, Mahlknecht U, Ho A, Schaich M, Kramer M (2012). Therapeutic platelet transfusion versus routine prophylactic transfusion in patients with haematological malignancies: an open-label, multicentre, randomised study. Lancet.

[11] Stanworth SJ, Estcourt LJ, Powter G, Kahan BC, Dyer C, Choo L, Bakrania L, Llewelyn C, Littlewood T, Soutar R (2013). A no-prophylaxis platelet-transfusion strategy for hematologic cancers. N Engl J Med.

[12] Rebulla P, Finazzi G, Marangoni F, Avvisati G, Gugliotta L, Tognoni G, Barbui T, Mandelli F, Sirchia G (1997). The threshold for prophylactic platelet transfusions in adults with acute myeloid leukemia. Gruppo Italiano Malattie Ematologiche Maligne dell'Adulto. N Engl J Med.

[13] Heckman KD, Weiner GJ, Davis CS, Strauss RG, Jones MP, Burns CP (1997). Randomized study of prophylactic platelet transfusion threshold during induction therapy for adult acute leukemia: 10,000/microL versus 20,000/microL. J Clin Oncol.

[14] Zumberg MS, del Rosario ML, Nejame CF, Pollock BH, Garzarella L, Kao KJ, Lottenberg R, Wingard JR (2002). A prospective randomized trial of prophylactic platelet transfusion and bleeding incidence in hematopoietic stem cell transplant recipients: 10,000/L versus 20,000/microL trigger. Biol Blood Marrow Transplant.

[15] Friedmann AM, Sengul H, Lehmann H, Schwartz C, Goodman S (2002). Do basic laboratory tests or clinical observations predict bleeding in thrombocytopenic oncology patients? A reevaluation of prophylactic platelet transfusions. Transfus Med Rev.

[16] Tanaka KA, Bolliger D, Vadlamudi R, Nimmo A (2012). Rotational thromboelastometry (ROTEM)-based coagulation management in cardiac surgery and major trauma. J Cardiothorac Vasc Anesth.

[17] Korpallova B, Samos M, Bolek T, Skornova I, Kovar F, Kubisz P, Stasko J, Mokan M (2018). Role of Thromboelastography and rotational Thromboelastometry in the Management of Cardiovascular Diseases. Clin Appl Thromb Hemost.

[18] Baryshnikova E, Di Dedda U, Ranucci M (2019). A comparative study of SEER Sonorheometry versus standard coagulation tests, rotational Thromboelastometry, and multiple electrode Aggregometry in cardiac surgery. J Cardiothorac Vasc Anesth.

[19] Toth O, Calatzis A, Penz S, Losonczy H, Siess W (2006). Multiple electrode aggregometry: a new device to measure platelet aggregation in whole blood. Thromb Haemost.

[20] Kirmani BH, Johnson RI, Agarwal S (2017). Platelet function testing in cardiac surgery: a comparative study of electrical impedance aggregometry and thromboelastography. Platelets.

[21] Shams Hakimi C, Singh S, Hesse C, Jeppsson A (2019). Effects of fibrinogen and platelet transfusion on coagulation and platelet function in bleeding cardiac surgery patients. Acta Anaesthesiol Scand.

[22] Hosokawa K, Ohnishi T, Kondo T, Fukasawa M, Koide T, Maruyama I, Tanaka KA (2011). A novel automated microchip flow-chamber system to quantitatively evaluate thrombus formation and antithrombotic agents under blood flow conditions. J Thromb Haemost.

[23] Kaikita K, Hosokawa K, Dahlen JR, Tsujita K (2019). Total Thrombus-formation analysis system (T-TAS): clinical application of quantitative analysis of Thrombus formation in cardiovascular disease. Thromb Haemost.

[24] Mitsuse T, Kaikita K, Ishii M, Oimatsu Y, Nakanishi N, Ito M, Arima Y, Sueta D, Iwashita S, Fujisue K (2020). Total Thrombus-formation analysis system can predict 1-year bleeding events in patients with coronary artery disease. J Atheroscler Thromb.

[25] Yamamoto K, Ito T, Nagasato T, Shinnakasu A, Kurano M, Arimura A, Arimura H, Hashiguchi H, Deguchi T, Maruyama I (2019). Effects of glycemic control and hypoglycemia on Thrombus formation assessed using automated microchip flow chamber system: an exploratory observational study. Thromb J.

[26] Daugirdas JT, Bernardo AA (2012). Hemodialysis effect on platelet count and function and hemodialysis-associated thrombocytopenia. Kidney Int.

[27] Paniccia R, Priora R, Liotta AA, Abbate R (2015). Platelet function tests: a comparative review. Vasc Health Risk Manag.

[28] Corada M, Mariotti M, Thurston G, Smith K, Kunkel R, Brockhaus M, Lampugnani MG, Martin-Padura I, Stoppacciaro A, Ruco L (1999). Vascular endothelial-cadherin is an important determinant of microvascular integrity in vivo. Proc Natl Acad Sci U S A.

[29] Dejana E, Tournier-Lasserve E, Weinstein BM (2009). The control of vascular integrity by endothelial cell junctions: molecular basis and pathological implications. Dev Cell.

[30] Malfait F (2018). Vascular aspects of the Ehlers-Danlos syndromes. Matrix Biol.

[31] Wylie LA, Mouillesseaux KP, Chong DC, Bautch VL (2018). Developmental SMAD6 loss leads to blood vessel hemorrhage and disrupted endothelial cell junctions. Dev Biol.

